# Long Non-coding Antisense RNA *DDIT4-AS1* Regulates Meningitic *Escherichia coli*-Induced Neuroinflammation by Promoting *DDIT4* mRNA Stability

**DOI:** 10.1007/s12035-021-02690-6

**Published:** 2022-01-05

**Authors:** Bo Yang, Bojie Xu, Ruicheng Yang, Jiyang Fu, Liang Li, Dong Huo, Jiaqi Chen, Xiaopei Yang, Chen Tan, Huanchun Chen, Xiangru Wang

**Affiliations:** 1 State Key Laboratory of Agricultural Microbiology, College of Veterinary Medicine, Huazhong Agricultural University, Wuhan, Hubei China; 2 Key Laboratory of Preventive Veterinary Medicine in Hubei Province, The Cooperative Innovation Center for Sustainable Pig Production, Wuhan, Hubei China; 3 Key Laboratory of Development of Veterinary Diagnostic Products, Ministry of Agriculture of the People’s Republic of China, Wuhan, Hubei China; 4 International Research Center for Animal Disease, Ministry of Science and Technology of the People’s Republic of China, Wuhan, Hubei China

**Keywords:** Antisense lncRNA, *DDIT4-AS1*, *DDIT4*, *Escherichia coli*, Neuroinflammation

## Abstract

Our previous studies have shown that meningitic *Escherichia coli* can colonize the brain and cause neuroinflammation. Controlling the balance of inflammatory responses in the host central nervous system is particularly vital. Emerging evidence has shown the important regulatory roles of long non-coding RNAs (lncRNAs) in a wide range of biological and pathological processes. However, whether lncRNAs participate in the regulation of meningitic *E. coli*-mediated neuroinflammation remains unknown. In the present study, we characterized a cytoplasm-enriched antisense lncRNA *DDIT4-AS1*, which showed similar concordant expression patterns with its parental mRNA *DDIT4* upon *E. coli* infection. *DDIT4-AS1* modulated *DDIT4* expression at both mRNA and protein levels. Mechanistically, *DDIT4-AS1* promoted the stability of *DDIT4* mRNA through RNA duplex formation. *DDIT4-AS1* knockdown and *DDIT4* knockout both attenuated *E. coli*-induced NF-κB signaling as well as pro-inflammatory cytokines expression, and *DDIT4-AS1* regulated the inflammatory response by targeting *DDIT4*. In summary, our results show that *DDIT4-AS1* promotes *E. coli*-induced neuroinflammatory responses by enhancing the stability of *DDIT4* mRNA through RNA duplex formation, providing potential nucleic acid targets for new therapeutic interventions in the treatment of bacterial meningitis.

**Supplementary Information:**

The online version contains supplementary material available at 10.1007/s12035-021-02690-6.

## Introduction

Bacterial meningitis is the most important life-threatening infection of the central nervous system (CNS) and continues to be a significant cause of mortality and morbidity [1, 2]. Despite advances in antimicrobial treatment, survivors suffer from neurological sequelae including cognitive impairment, developmental delay, and hearing loss [1, 3]. *Escherichia coli* (*E. coli*) is the most common Gram-negative bacillary organism that causes meningitis in neonates and children, and hematogenous spread is the leading spreading way of *E. coli* meningitis [4, 5]. Our earlier studies have shown that meningitic *E. coli* can colonize the brain and cause neuroinflammation [6, 7]. However, how host respond to invading bacteria and modulate neuroinflammatory responses are still poorly understood.

In recent years, there has been increasing interest in long non-coding RNAs (lncRNAs). These are defined as RNAs longer than 200 nucleotides in length with no protein-coding capacity [8] and can be further classified as antisense lncRNAs, long intergenic noncoding RNAs (lincRNAs), intronic lncRNAs, and enhancer RNAs (eRNAs) based on their genome position [9]. Accumulating evidence has shown that lncRNAs play significant regulatory roles in diverse biological processes [10]. Further, they have been proposed to perform their functions through diverse mechanisms, including binding with RNA or DNA through nucleic acid base pairing, interacting with proteins through higher-order RNA structures [9, 11, 12]. However, our knowledge about the function and the potential molecular regulatory mechanisms of lncRNAs in bacterial meningitis is still limited.

DNA damage inducible transcript 4 (*DDIT4*), also known as *REDD1*/*RTP801*/*Dig2*, was originally characterized by its transcriptional upregulation in response to DNA damage. *DDIT4* is an inhibitor of mammalian target of rapamycin (mTOR) and is induced by multiple cellular stresses including hypoxia, heat shock, energy depletion, starvation, and LPS [13, 14]. *DDIT4* participates in regulating a broad spectrum of cellular and biological functions, such as cell survival, growth, apoptosis, and autophagy [15, 16]. Importantly, a growing body of evidence suggests that *DDIT4* plays a crucial role in inflammation [14, 17–20]. Nevertheless, the function of *DDIT4* in the CNS remains poorly characterized, especially in the context of bacterial infection.

In the present study, we characterized *DDIT4-AS1*, a long non-coding antisense transcript for *DDIT4*, is a cytoplasm-enriched antisense lncRNA and showed similar concordant expression patterns with *DDIT4* upon *E. coli* infection. In addition, *DDIT4-AS1* was found to positively regulate *DDIT4* expression by promoting the stability of *DDIT4* mRNA through RNA duplex formation. Decreasing the expression of *DDIT4-AS1* or *DDIT4* attenuated *E. coli*-induced pro-inflammatory factors production and NF-κB signaling. Moreover, we demonstrated that *DDIT4-AS1* regulates the inflammatory response by targeting *DDIT4*. Taken together, these findings reveal that *DDIT4-AS1* regulates meningitic *E. coli*-induced neuroinflammation by promoting *DDIT4* mRNA stability, providing novel nucleic acid targets for future prevention and treatment of bacterial meningitis.

## Materials and Methods

### Bacterial Strains

The meningitic *E. coli* strain PCN033 used herein is a highly virulent cerebrospinal fluid isolate, originally isolated in Hunan Province, China, in 2006 [21]. The PCN033 strain was routinely grown aerobically at 37 °C in Luria–Bertani (LB) medium. The strain was evidenced to be capable of causing host blood–brain barrier (BBB) disruption and severe neuroinflammation in vitro and in vivo [6].

### Cell Culture and Infection

The human astrocyte cell line U251 was cultured in Dulbecco’s modified Eagle’s medium (DMEM) supplemented with 10% heat-inactivated fetal bovine serum (FBS). Human brain microvascular endothelial cells (hBMECs) were cultured in RPMI 1640 medium supplemented with 10% FBS, 2 mM L-glutamine, 1 mM sodium pyruvate, essential amino acids, nonessential amino acids, vitamins, penicillin, and streptomycin (100 U/mL). The human microglia cell line HMO6 was cultured in DMEM supplemented with 10% heat-inactivated FBS. All cells were incubated in a 37 °C incubator with a 5% CO_2_ atmosphere until monolayer confluence. Confluent cells were washed three times with phosphate-buffered saline (PBS) and starved in serum-free medium for 16–18 h prior to infection. Overnight cultures of PCN033 were resuspended and diluted in serum-free medium and then added to the starved monolayer cells at a multiplicity of infection (MOI) of 10.

### Reagents

The DDIT4 (rabbit) antibody (#10,638–1-AP, 1:1000 dilution) and β-actin (mouse) antibody (#66,009–1-Ig, 1:5000 dilution) were obtained from Proteintech (Chicago, IL, USA). Anti-NF-κB p65 (#6956, 1:1000 dilution) and anti-phospho-p65 (#3033, 1:1000 dilution) were obtained from Cell Signaling Technology (Danvers, MA, USA). Cy3-labeled goat anti-mouse (#A0521, 1:200 dilution) and FITC-labeled goat anti-rabbit antibodies (#A0562, 1:200 dilution) and DAPI were obtained from Beyotime Institute of Biotechnology (China). The *DDIT4-AS1* antisense oligonucleotides (ASO) and the control ASO were purchased from Integrated Biotech Solutions Co., Ltd. (Shanghai, China); the sequences are listed in Table S2. The clustered regularly interspaced short palindromic repeats (CRISPR)/Cas9 plasmid pYSY-spCas9-sgRNA-Puro was obtained from YSY Biotech (Nanjing, China). The transfection reagent jetPRIME was purchased from Polyplus Transfection (Illkirch, France). The RNA polymerase II transcription inhibitor α-amanitin was purchased from Medchem Express (Princeton, NJ, USA). RNAse A + T cocktail was purchased from Thermo Fisher Scientific (Waltham, MA, USA). The super electrochemiluminescence (ECL) kit was obtained from US Everbright Inc. (Suzhou, China).

### RNA Extraction and Quantitative Real-Time PCR (qPCR)

Total RNA was extracted from astrocytes U251 cells using TRIzol® Reagent (Aidlab Biotech, Beijing, China) in accordance with the manufacturer’s protocol. One microgram of the total RNA was subjected to cDNA synthesis using HiScript II Q RT SuperMix (Vazyme, Nanjing, China). Real-time PCR was performed with the MonAmp™ SYBR Green qPCR Mix (RN04005M, Monad Biotech Co., Ltd, Wuhan, China) according to the manufacturer’s instructions. The transcriptional levels of all mRNA targets were normalized to *GAPDH*. Primers for the quantitative real-time PCR are listed in Table S1. Each assay was performed in triplicate.

### *RNA Fluorescence *In Situ* Hybridization (FISH)*

Cy3-labeled *DDIT4-AS1* and Fam-labeled *DDIT4* FISH probes were obtained from Genscript (Nanjing, China) and the sequences are listed in Table S2. Briefly, the astrocytes were fixed with 4% paraformaldehyde and then treated with 1% Triton X-100 in PBS, followed by hybridization with probes targeting *DDIT4-AS1* and *DDIT4*, respectively. The cells were counterstained with DAPI and the fluorescence signals were visualized under a fluorescent microscope.

### Nuclear and Cytoplasmic Fractionation

The experiment was performed as previously described [22]. Briefly, the astrocytes were treated with Trypsin–EDTA, resuspended in DMEM, centrifuged, and resuspended in hypotonic buffer (10 mM Hepes, pH 7.9, 1.5 mM MgCl2, 10 mM KCl). Subsequently, 10% Nonidet P-40 was added to the samples, which were then centrifuged for 7 min, and the supernatant was collected as the cytoplasm extracts. The remainder was considered as nuclear fraction and was subjected to washing four times with hypotonic buffer.

### Overexpression/Knockdown Experiments

To construct a *DDIT-AS1* overexpression plasmid, the full-length *DDIT4-AS1* sequence was amplified from U251 cDNA by overlap-expression PCR and subsequently cloned into the pCDNA3.1 ( +) vector. The plasmids were transfected with jetPRIME. For the knockdown of *DDIT-AS1*, 300 nM *DDIT4-AS1* ASO was transfected into astrocytes using jetPRIME.

### Plasmid Constructs

pEGFP-N1-MUT was generated by mutating the start codon ATGGTG to ATTGTT in pEGFP-N1 vector using overlapping extension PCR. The primers were listed in Table S1. The DDIT4-AS1-ORF-pEGFP-N1-MUT and DDIT4-pEGFP-N1-MUT plasmids were synthesized by Genscript (Nanjing, China). In brief, the DDIT4-AS1 ORF sequence and DDIT4 CDS sequence with the stop codon removed were cloned into the pEGFP-N1-MUT.

### RNA Antisense Purification (RAP)

We designed and synthesized five RAP probes targeting *DDIT4-AS1*, and each DNA oligonucleotide probe was modified with a 5′ biotin. The sequences of the probes are presented in Table S2. RAP was performed using the RAP Kit (BersinBio, Guangzhou, China). According to the manufacturer’s instruction, approximately 4 × 10^7^ cells were crosslinked with 1% formaldehyde. The crosslinked cells were solubilized using lysis buffer with protease inhibitor and RNase inhibitor, followed by DNA elimination using DNase. The solubilized lysates were subsequently incubated with the prepared *DDIT4-AS1* probe mixture or the control probes and then immobilized with streptavidin-coated magnetic beads. Beads with captured hybrids were washed five times with washing buffer. RNA elution buffer was added to release the RNA from the beads and proteinase K was used to remove all proteins. Finally, RNA was isolated and subjected to qPCR assays.

### RNase Protection Assay

The procedure was performed as previously described [23]. Two sets of primers were used. The first targeted the overlapping (OL) region of *DDIT4* and *DDIT-AS1*, and the second targeted the non-OL region of *DDIT4* mRNA. The RNA samples from astrocytes were incubated at 37 °C for 1 h and then treated with RNAse A + T cocktail at 37 °C for 30 min. The samples were then treated with proteinase K at 50 °C for 1 h, followed by RNA purification using a MicroElute RNA Clean-up Kit (Norcross, GA, USA). Subsequently, the purified RNAs were subjected to cDNA synthesis and PCR amplification to detect the OL and non-OL regions of *DDIT4* mRNA, using two distinct sets of primers.

### α-Amanitin Treatment

The astrocytes were transfected with the *DDIT4-AS1* overexpression plasmid or control plasmid. After 24 h, these cells were treated with 5 μM α-amanitin for 6 h and then the cells were harvested for RNA extraction and qPCR. Three independent samples were obtained from each group.

### CRISPR/Cas9 Genomic Editing

Two sgRNAs targeting human *DDIT4* were cloned into the all-in-one vector to generate the pYSY-spCas9-DDIT4-sgRNA-Puro plasmids. Astrocytes were transfected with the two CRISPR/Cas9 plasmids using jetPRIME. The cells were incubated at 37 °C with 5% CO_2_ for 24 h, and then, 400 ng/mL puromycin was added and incubated for another 48 h. The surviving cells were then transferred into 96-well plates with limiting dilution and incubated at 37 °C with 5% CO_2_ until a single-cell clone was formed. Genomic DNA from each cell clone was extracted using the QuickExtract DNA Extraction Solution (YSY Biotech, Nanjing, China). PCR was performed to amplify the target region with the following primers: 5′-CTTACAGCGGCTTCTACGC-3′ (forward) and 5′-GGCTCTGACCCCTTCCAG-3′ (reverse). Finally, the positive editing cells were validated by sequencing.

### Western Blotting

The astrocytes were lysed in radio immunoprecipitation assay (RIPA) buffer with a protease inhibitor cocktail (Sigma-Aldrich, USA) and then centrifuged at 12,000 rpm for 10 min at 4 °C. A BCA protein assay kit (Beyotime, China) was used to measure the protein concentration in the supernatant and the cell lysates were then subjected to western blot analyses as previously described [6]. The blots were visualized with ECL reagents.

### Immunofluorescence Microscopy

The astrocytes were washed with PBS three times and fixed with 4% paraformaldehyde. The fixed cells were treated with 1% Triton X-100 in PBS and blocked in 5% BSA in Tris-buffered saline with Tween 20 (TBST), and then incubated with the primary antibody. Herein, DDIT4 was labeled with FITC and p65 was labeled with Cy3. The cells in the dishes were mounted and visualized under a fluorescence microscope.

### Statistical Analysis

Data are expressed as the mean ± SD and the significance of differences between groups was evaluated by unpaired two-tail *t*-test or one-way analysis of variance (ANOVA) embedded in GraphPad Prism, version 7.0 (GraphPad Software Inc., La Jolla, CA, USA). A level of *p* < 0.05 (*) was considered significant, and *p* < 0.01 (**) or *p* < 0.001 (***) was considered extremely significant.

## Results

### Antisense lncRNAs Display Differential Expression upon Meningitic *E. coli* Infection

**Fig. 1:**
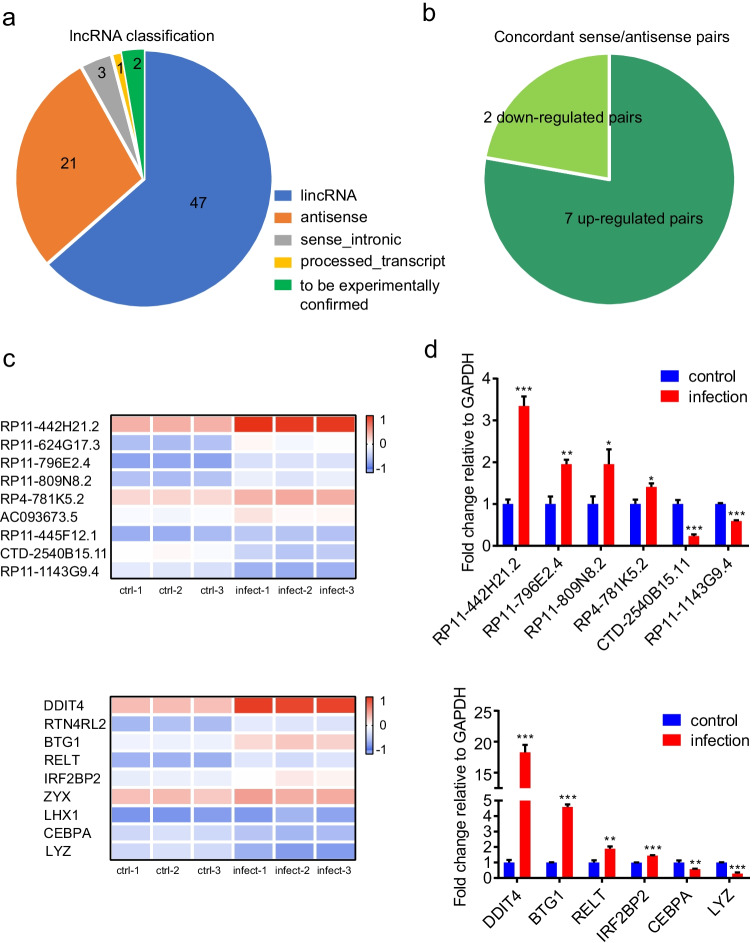
Antisense lncRNAs display differential expression upon meningitic *Escherichia coli* infection. **a** Genomic classification of differentially expressed lncRNAs. **b** Number of concordant upregulated and downregulated sense/antisense pairs in the *E. coli*-infected astrocytes. **c** Heatmap representing the nine concordant differentially expressed lncRNA–mRNAs pairs. **d** qPCR verification of the concordant differentially expressed lncRNA–mRNAs pairs. *GAPDH* was used as an internal control. Data are presented as the mean ± SD from three independent experiments; *p* values were analyzed by unpaired two-tail *t*-test; *p < 0.05, **p < 0.01, ***p < 0.001

We have previously performed lncRNAs sequencing in meningitic *E. coli*-infected astrocytes [24]. The expression profiling data revealed that 74 lncRNAs were differentially expressed, including 45 upregulated and 29 downregulated. In addition, the expression of 2045 mRNAs was significantly changed upon *E. coli* infection, of which 1150 were upregulated and 895 were downregulated. Subgroup analysis showed genomic classification of differentially expressed lncRNAs in Fig. 1a; lincRNAs represented the largest category (63.5%) of all differentially expressed lncRNAs, followed by antisense lncRNAs, which accounted for 28.4% (Table S4). A growing body of evidence suggests that antisense lncRNAs are frequently functional and regulate the expression of their sense protein-coding RNAs through diverse regulatory mechanisms, including transcription-related modulation, RNA–DNA interactions, and RNA–RNA interactions [25, 26]. To characterize the role of antisense lncRNAs in the pathological process of meningitic *E. coli* infection, we evaluated the expression of 21 antisense lncRNAs and their corresponding protein-coding mRNAs. We found that nine lncRNA–mRNA pairs were differentially expressed upon *E. coli* infection, and all of them showed concordant patterns of expression, including seven upregulated pairs (*RP11-442H21.2*/*DDIT4*, *RP11-624G17.3*/*RTN4RL2*, *RP11-796E2.4*/*BTG1*, *RP11-809N8.2*/*RELT*, *RP4-781K5.2*/*IRF2BP2*, *AC093673.5*/*ZYX*, and *RP11-445F12.1*/*LHX1*) and 2 downregulated pairs (*CTD-2540B15.11*/*CEBPA* and *RP11-1143G9.4*/*LYZ*) (Fig. 1b and c) (Table S5). Quantitative real-time PCR was performed for verification of differentially expressed lncRNA–mRNA pairs. As shown in Fig. 1d, six lncRNA–mRNA pairs displayed concordant expression, among which the *RP11-442H21.2*/*DDIT4* pair showed the most significant difference. Combined with the fact that *RP11-442H21.2* was highly expressed, as compared with other differentially expressed lncRNAs, and that *DDIT4* has been reported to be involved in inflammation, the *RP11-442H21.2*/*DDIT4* pair was chosen for further study.

### *DDIT4-AS1* Is a Cytoplasm-Enriched Antisense lncRNA

**Fig. 2:**
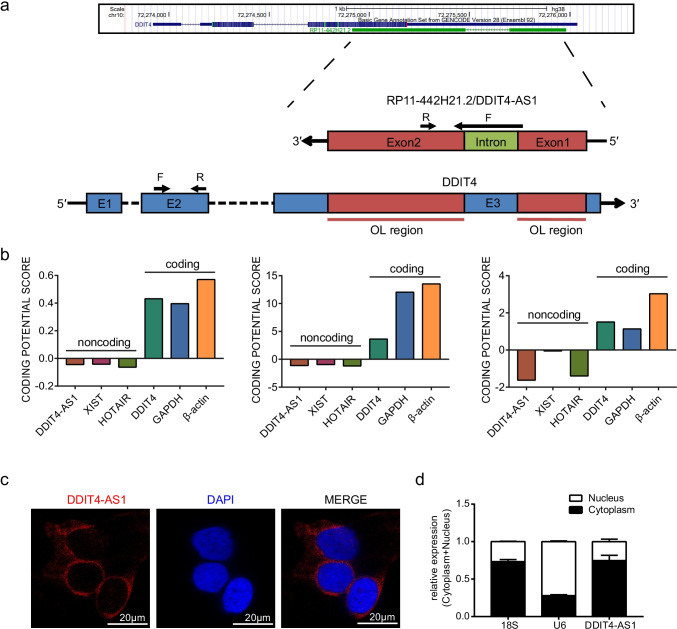
*DDIT4-AS1* is a cytoplasm-enriched antisense lncRNA. **a** Schematic illustration of the genome organization of *DDIT4* and *DDIT4-AS1* at locus chr10 (q22.1). Arrows show the transcription direction. The qPCR primers of *DDIT4* and *DDIT4-AS1* are shown in the schema. The overlapping (OL) regions are also indicated in the schema. **b** Coding potential of six RNAs (*DDIT4-AS1*, *XIST*, *HOTAIR*, *DDIT4*, *GAPDH*, and *β-actin*) predicted by CNCI, CPC, and PLEK. **c** Representative images of RNA FISH showing cytoplasm localization of *DDIT4-AS1* (red) in astrocytes. The cell nucleus was stained in blue with DAPI. Scale bar: 20 μm. **d** Subcellular localization of *DDIT4-AS1* in cytoplasm and nucleus (*n* = 3). *18 s* mRNA and U6 mRNA were controls for cytoplasmic and nuclear RNAs, respectively

Based on the UCSC genome browser, *RP11-442H21.2* is located at chromosomal band 10q22.1 and consists of two exons with a full length of 847 nt. *RP11-442H21.2* was identified as a single antisense lncRNA transcribed from the reverse strand of the *DDIT4* locus; therefore, it is also named *DDIT4-AS1*. The whole sequence of *DDIT4-AS1* is shown in Table S3. As shown in Fig. 2a, the full length of *DDIT4-AS1* shares a reverse complement region with the third exon of *DDIT4*, which is an 847 nt long sequence that we referred to as the overlapping (OL) region. To exclude the coding potential of *DDIT4-AS1*, three tools, including Coding-Non-Coding Index (CNCI) [27], Coding Potential Calculator (CPC) [28], and Predictor of Long Non-coding RNAs and Messenger RNAs Based on K-mer Scheme (PLEK) [29] were utilized to perform coding-potential analysis. Two well-known lncRNAs, *XIST* and *HOTAIR*, as well as three mRNAs, *DDIT4*, *GAPDH*, and *β-actin*, were used as controls. It is apparent from Fig. 2b that *DDIT4-AS1* had a very low coding potential and comparable to *XIST* and *HOTAIR*. In addition, we predicted a short 141-nt small ORF in *DDIT4-AS1* with the potential to encode peptide; the sequence was shown in Table S3. A series of constructs were generated to further validate the coding potential of *DDIT4-AS1* (Fig. S1a). pEGFP-N1-MUT was generated by mutating the start codon ATGGTG to ATTGTT in pEGFP-N1 vector. The *DDIT4-AS1* ORF sequence and *DDIT4* CDS sequence were cloned into the pEGFP-N1-MUT. As expected, substantial expression of the EGFP was observed in pEGFP-N1-WT-transfected cells, while mutation of the start codon abolished the expression of the EGFP protein. Importantly, the expression of the EGFP was observed in DDIT4-pEGFP-N1-MUT-transfected cells, but not in DDIT4-AS1-ORF-pEGFP-N1-MUT-transfected cells, which further proved the non-coding feature of *DDIT4-AS1* (Fig. S1b). In order to investigate the subcellular localization of *DDIT4-AS1*, a FISH assay was conducted, and the results showed that *DDIT4-AS1* was enriched in the cytoplasm of astrocytes (Fig. 2c). This result was further confirmed by the quantification of nucleus/cytoplasm RNAs (Fig. 2d). Collectively, these data indicate that *DDIT4-AS1* is a cytoplasm-enriched antisense lncRNA.

### *DDIT4-AS1* and *DDIT4* Are Upregulated upon Meningitic *E. coli* Infection

**Fig. 3:**
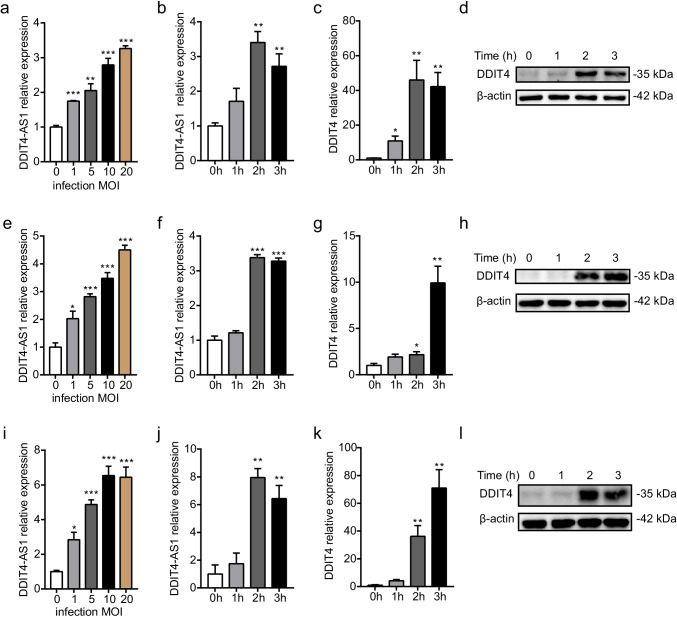
*DDIT4-AS1* and *DDIT4* show similar concordant expression patterns upon meningitic *E. coli* infection. **a**, **b** The human astrocyte cell line U251 was infected with meningitic *E. coli* at the indicated multiplicity of infection (MOI) for 3 h or an MOI of 10 for the indicated times. The expression of *DDIT4-AS1* was detected by qPCR. **c**, **d** The mRNA and protein expression of *DDIT4* in U251 cells in response to *E. coli* infection at an MOI of 10. **e**–**h** Expression of *DDIT4-AS1* and *DDIT4* in *E. coli*-infected human brain microvascular endothelial cells (hBMEC). **i–l** Expression of *DDIT4-AS1* and *DDIT4* in *E. coli*-infected human microglia cell line HMO6. *GAPDH* was used as the reference control for qPCR. Data represent the mean ± SD (*n* = 3/group). Statistical analysis was carried out by one-way ANOVA. *p* < 0.05 (*), *p* < 0.01 (**), *p* < 0.001 (***)

We next examined the expression of *DDIT4-AS1* and *DDIT4* upon meningitic *E. coli* infection. To prevent non-specific amplification, the forward primer of *DDIT4-AS1* was designed to span the first and second exons. The primers of *DDIT4* were designed on the second exon to distinguish *DDIT4-AS1* and *DDIT4*. We first analyzed the level of *DDIT4-AS1* in the *E. coli*-infected astrocyte cell line U251 using qPCR. The results showed that the expression of *DDIT4-AS1* was significantly increased in a dose- and time-dependent manner (Fig. 3a, b). In addition, the mRNA and protein levels of *DDIT4* also elevated in a time-dependent manner (Fig. 3c, d). Furthermore, we detected the expression of *DDIT4-AS1* and *DDIT4* in human brain microvascular endothelial cells (hBMECs) and microglia cell line HMO6. The results were similar to those observed in *E. coli*-infected astrocytes; *E. coli* infection induced the notable upregulation of *DDIT4-AS1* and *DDIT4* in hBMECs (Fig. 3e–h) and microglia (Fig. 3i–l). The similar concordant expression patterns of *DDIT4-AS1* and *DDIT4* indicated a strong correlation between them. Given that astrocytes and hBMECs are the two major BBB cell types and microglia are considered the major inflammatory cell type in the CNS, the *DDIT4-AS1*/*DDIT4* pair might play an important role in the CNS upon *E. coli* infection.

### *DDIT4-AS1* Positively Regulates *DDIT4* Expression

**Fig. 4:**
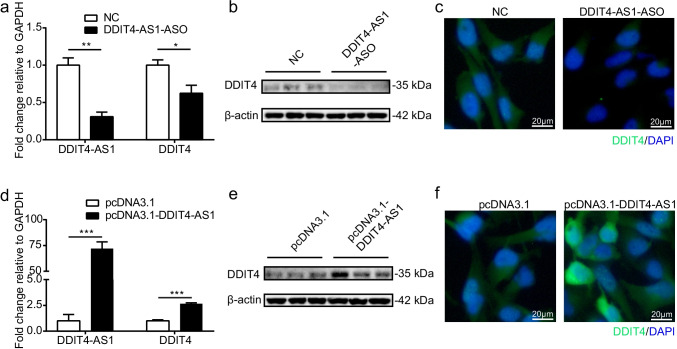
*DDIT4-AS1* regulates *DDIT4* expression at the mRNA and protein levels. **a**–**c** U251 cells were transfected with modified antisense oligonucleotides (ASO) targeting *DDIT4-AS1*, and the expression of *DDIT4* was detected by qPCR, western blotting (WB), and immunofluorescence (IF) microscopy. **d**–**f** U251 cells were transfected with *DDIT4-AS1* overexpression plasmid, and the expression of *DDIT4* was detected by qPCR, WB, and IF. For the qPCR experiment, *GAPDH* was used as the reference control. Data represent the mean ± SD (*n* = 3/group). *p* < 0.05 (*), *p* < 0.01 (**), *p* < 0.001 (***). For the IF experiment, *DDIT4* was labeled in green, and the cell nucleus was stained in blue with DAPI. Scale bar indicates 20 μm

To determine whether *DDIT4-AS1* can regulate the expression of *DDIT4*, we transiently downregulated it in astrocytes using modified ASO, which is a single-strand RNA targeting *DDIT4-AS1* without directly affecting the expression of *DDIT4*. *DDIT4-AS1*-depleted cells showed decreased *DDIT4* mRNA levels (Fig. 4a). In addition, western blot and immunofluorescence results showed that DDIT4 protein levels were also reduced in *DDIT4-AS1*-depleted cells (Fig. 4b, c). We also overexpressed *DDIT4-AS1* by transfecting the full-length sequence of *DDIT4-AS1* in astrocytes; as we expected, overexpression of *DDIT4-AS1* induced increased mRNA and protein expression of *DDIT4* (Fig. 4d–f). These findings indicate that *DDIT4-AS1* positively regulates *DDIT4* expression.

### *DDIT4-AS1* Increases* DDIT4* mRNA Stability by Forming an RNA Duplex

**Fig. 5:**
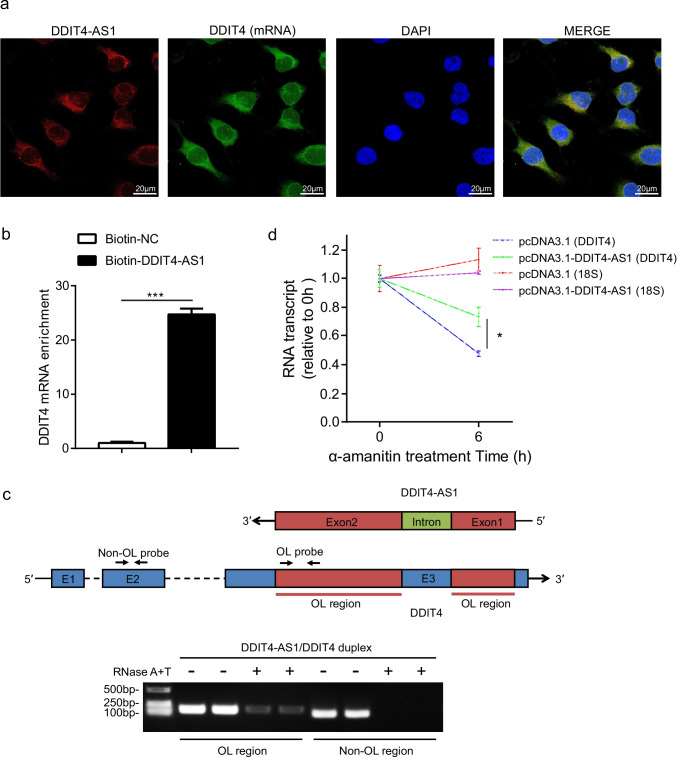
*DDIT4-AS1* forms a duplex RNA–RNA structure with *DDIT4*. **a** Representative images of RNA FISH showing co-localization of *DDIT4-AS1* (red) with *DDIT4* mRNA (green) in the cytoplasm. The cell nucleus was stained in blue with DAPI. Scale bar indicates 20 μm. **b** qPCR was used to measure the enrichment of *DDIT4* mRNA in biotin-labeled *DDIT4-AS1* pull-down samples compared to that in the negative control. **c** RNase protection assay was performed on RNA samples from U251 cells, and PCR amplification was used to detect the overlapping (OL) and the non-OL regions of *DDIT4*. **d** U251 cells were transfected with the *DDIT4-AS1* overexpression plasmid or the empty vector for 24 h and then treated with 5 μM α-amanitin for 6 h. The expression of *18S* and *DDIT4* was determined by qPCR. Error bars in **b** and **d** represent the mean ± SD (*n* = 3/group). *p* < 0.05 (*), *p* < 0.001 (***)

Based on the reverse complement nucleotides between *DDIT4-AS1* and *DDIT4*, we speculated that *DDIT4-AS1* and *DDIT4* could form an RNA duplex to increase the stability of *DDIT4* mRNA. To test our hypothesis, we first examined the cellular localization of *DDIT4-AS1* and *DDIT4* by FISH. As shown in Fig. 5a, some fluorescence signals of *DDIT4-AS1* and *DDIT4* overlapped, implying that *DDIT4-AS1* is likely to interact with *DDIT4* in the cytoplasm. To further verify the direct interaction between *DDIT4-AS1* and *DDIT4*, RNA antisense purification (RAP) was conducted using biotin-labeled RNA probes targeting *DDIT4-AS1*. We observed that *DDIT4* mRNA was significantly enriched in biotin-labeled *DDIT4-AS1* pull-down samples compared to levers in negative control (Fig. 5b). In addition, we used an RNase protection assay (RPA) on RNA from astrocytes to confirm the formation of the RNA duplex. The detection probes were designed on the overlapping (OL) and non-OL regions of *DDIT4*. PCR amplification results showed that the non-OL region was completely digested by RNase, whereas the OL region was partially protected from degradation (Fig. 5c). We next evaluated the effect of *DDIT4-AS1* on the stability of *DDIT4* by blocking new RNA synthesis with the RNA polymerase II transcription inhibitor α-amanitin over a 6-h period. As shown in Fig. 5d, 18 s ribosomal RNA, a product of RNA polymerase I, showed no significant changes upon α-amanitin treatment. Approximately 60% of *DDIT4* mRNA was consumed after 6 h, whereas the expression of *DDIT4* was partially restored by overexpressing *DDIT4-AS1*, which revealed that the stability of *DDIT4* was elevated by *DDIT4-AS1*. Taken together, these data demonstrate that *DDIT4-AS1* and *DDIT4* could form an RNA duplex to increase *DDIT4* mRNA stability.

### Knockdown of *DDIT4-AS1* Suppresses *E. coli*-Induced Pro-inflammatory Factors Production and NF-κB Signaling

**Fig. 6:**
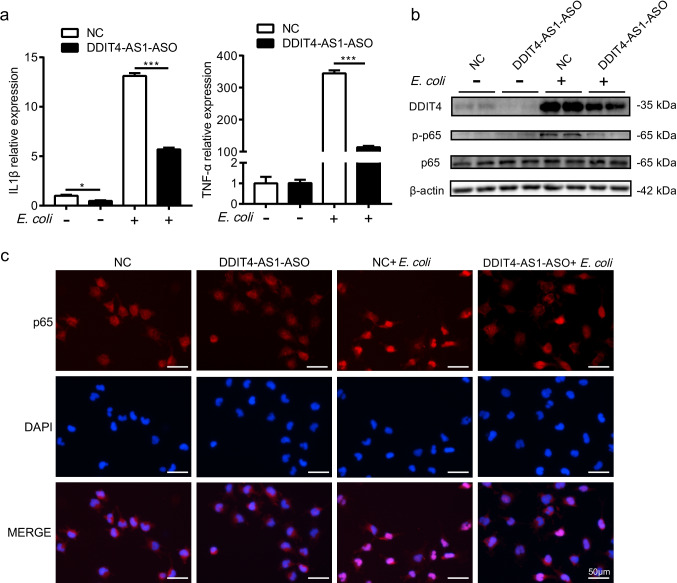
Knockdown of *DDIT4-AS1* suppresses *E. coli*-induced pro-inflammatory factors production and NF-κB signaling. **a** U251 cells were transfected with 300 nM *DDIT4-AS1* antisense oligonucleotide (ASO) or negative control for 24 h and then infected with *E. coli* at an MOI of 10 for 3 h. The expression of *IL-1β* and *TNF-α* was determined by qPCR. *GAPDH* was used as the internal reference. Data represented the mean ± SD (*n* = 3/group). Statistical analysis was carried out by one-way ANOVA. *p* < 0.001 (***). **b** U251 cells were transfected as in **a** and then infected with *E. coli* at an MOI of 10 for 2 h, and the protein levels of *DDIT4*, NF-κB p65, and phosphorylated p65 were determined by western blotting. **c** U251 cells were transfected as in **a** and then infected with *E. coli*; the translocation of the p65 subunit was detected by immunofluorescence microscopy. The p65 was labeled in red, and the cell nucleus was stained in blue with DAPI. Scale bar: 50 μm

Considering that *DDIT4* has been implicated in the regulation of inflammatory responses and *DDIT4-AS1* can positively regulate *DDIT4* expression, we next examined the effect of *DDIT4-AS1* knockdown on pro-inflammatory gene expression in astrocytes. As shown in Fig. 6a, *DDIT4-AS1* knockdown significantly inhibited *E. coli*-induced pro-inflammatory gene expression, including *IL-1β* and *TNF-α*. NF-κB-mediated pro-inflammatory gene expression plays a crucial role in the innate immune response against bacterial infection; thus, we evaluated the effect of *DDIT4-AS1* knockdown on NF-κB signaling. *E. coli* infection stimulated the phosphorylation of p65, and this effect was attenuated by the knockdown of *DDIT4-AS1* (Fig. 6b). We further examined the effect of *DDIT4-AS1* on NF-κB signaling using immunofluorescence microscopy. As expected, *E. coli* infection promoted p65 translocation from the cytoplasm to the nucleus, which was partly prevented by *DDIT4-AS1* knockdown (Fig. 6c). Collectively, these results suggest that *DDIT4-AS1* regulates pro-inflammatory factors production and NF-κB signaling.

### *DDIT4-AS1* Regulates the Inflammatory Response by Targeting *DDIT4*

**Fig. 7:**
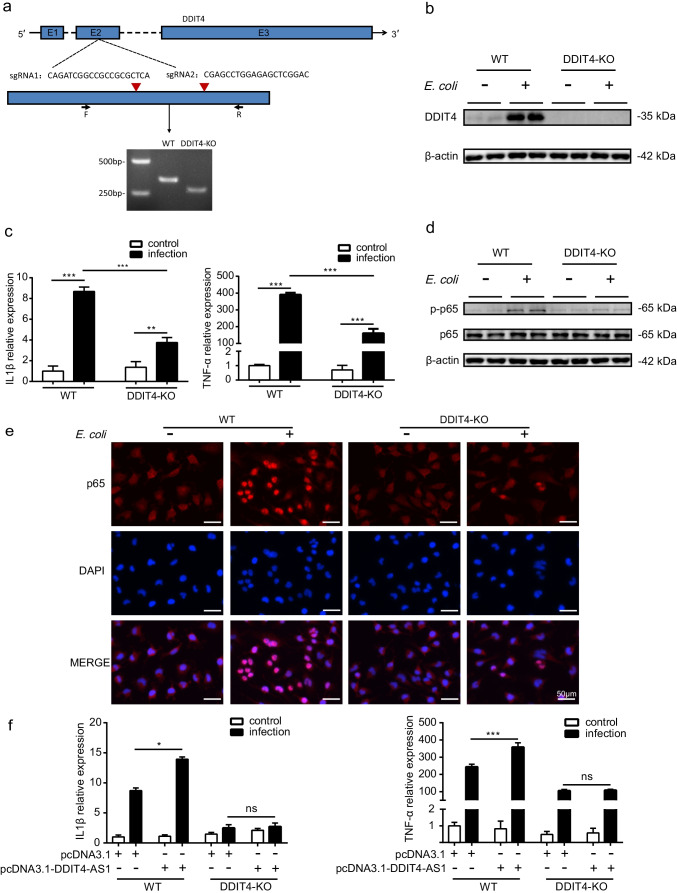
*DDIT4-AS1* regulates inflammatory response by targeting *DDIT4*. **a** Two small guide RNAs (sgRNA1 and sgRNA2) were designed to target the second exon of *DDIT4*, and PCR was used to identify the DDIT4 knockout (KO) using specific primers (F and R). **b** The *DDIT4*-KO cells and the wild-type (WT) cells were infected with *E. coli* at an MOI of 10 for 3 h; the expression of *DDIT4* was measured by western blotting. **c** qPCR was used to measure the expression of *IL-1β* and *TNF-α* in *E. coli*-infected *DDIT4*-KO cells and WT cells. **d** Western blot analysis of p65 phosphorylation in *DDIT4*-KO cells and WT cells upon *E. coli* infection. **e** Translocation of the p65 subunit was detected in *DDIT4*-KO cells and WT cells. The p65 was labeled in red, and the cell nucleus was stained in blue with DAPI. Scale bar: 50 μm. **f** The *DDIT4*-KO cells and WT cells were transfected with the *DDIT4-AS1* overexpression plasmid and then infected with *E. coli* for 3 h, and the expression of *IL-1β* and *TNF-α* was determined by qPCR. *GAPDH* was used as the internal reference. Data represented the mean ± SD (*n* = 3/group). Statistical analysis was carried out by one-way ANOVA. *p* < 0.05 (*), *p* < 0.01 (**), *p* < 0.001 (***)

To further verify that *DDIT4-AS1* regulates the inflammatory response by targeting *DDIT4*, we evaluated the function of *DDIT4* through deletion using the CRISPR/Cas9 approach. Two small guide RNAs were designed to target exon 2 of *DDIT4*, and the deletion was validated by PCR amplification (Fig. 7a). In addition, *E. coli* infection led to the upregulation of *DDIT4*, which was abolished by *DDIT4* deletion, with no *DDIT4* expression in the knockout (KO) cells (Fig. 7b). *DDIT4* knockout markedly suppressed *E. coli*-induced pro-inflammatory *IL-1β* and *TNF-α* expression, as well as infection-induced NF-κB p65 phosphorylation and nuclear translocation (Fig. 7c–e). Moreover, the overexpression of *DDIT4-AS1* augmented *E. coli*-induced *IL-1β* and *TNF-α* expression; however, the pro-inflammatory effect of *DDIT4-AS1* vanished in *DDIT4* KO cells (Fig. 7f), which revealed that *DDIT4-AS1* functions are mediated by *DDIT4*. Collectively, these data indicate that *DDIT4-AS1* plays a pro-inflammatory role in the progress of *E. coli* infection by promoting *DDIT4* mRNA stability.

## Discussion

A growing body of evidence suggests that lncRNAs are involved in a wide range of biological functions and lncRNAs are now emerging as important regulators of inflammation [30]. Antisense lncRNAs are defined as long non-coding RNAs from the opposite strand of the sense transcript of either protein-coding or nonprotein-coding genes [26, 31]. Antisense lncRNAs have been reported to modulate almost every level of gene regulation, including pre-transcriptional, transcriptional, and post-transcriptional gene regulatory mechanisms, to exert a broad spectrum of biological functions [25]. Importantly, antisense lncRNAs can act as positive and negative regulators of the corresponding sense transcript [31–33]. In the current study, we sought to explore the involvement of lncRNAs in the regulation of meningitic *E. coli*-mediated neuroinflammation. We focused our attention on antisense lncRNAs and selected the most significantly upregulated lncRNA–mRNA pair (*DDIT4-AS1*/*DDIT4*) for further studies. We found that the cytoplasm-enriched antisense lncRNA *DDIT4-AS1* showed concordant expression patterns with *DDIT4* upon *E. coli* infection, and *DDIT4-AS1* modulated *DDIT4* expression by enhancing the stability of *DDIT4* mRNA through RNA duplex formation, thereby promoting NF-κB activation and pro-inflammatory gene expression.

Meningitic *E. coli* infection of the host CNS relies on intricate interactions between the host BBB and bacteria. Our previous studies have characterized the transcriptome profiles of astrocytes in response to infection and revealed that lncRNAs are likely involved in the development of bacterial meningitis [24]. Indeed, the role of lncRNAs in the host cell response to bacterial infections has received increased attention in recent years, and studies show that lncRNAs actively respond to various bacterial infections, including *Salmonella*, *Helicobacter pylori*, *Mycobacterium*, and *Listeria monocytogenes* [34–38]. Interestingly, many lncRNAs have been reported to modulate inflammatory responses in the progress of bacterial infection. For example, an intergenic lncRNA *lincRNA-EPS* is downregulated in macrophages exposed to *L. monocytogenes* infection, which acts as a repressor of inflammatory responses by interacting with heterogeneous nuclear ribonucleoprotein L [39]. In contrast, the antisense lncRNA *AS-IL1α*, which is partially complementary to *IL-1α*, is upregulated following *L. monocytogenes* infection. *AS-IL1α* recruits RNA polymerase II to the IL-1α promoter, thereby enhancing IL1α expression [38]. In this study, we identified an *E. coli* infection-induced antisense lncRNA *DDIT4-AS1* that could promote NF-κB signaling by upregulating *DDIT4* expression. Our study further confirmed the important regulatory role of lncRNAs in the process of bacterial infection, which might provide potential new targets for future prevention of pathogenic *E. coli* meningitis.

We observed that *DDIT4-AS1* and *DDIT4* showed concordant expression patterns upon *E. coli* infection and *DDIT4-AS1* positively modulated *DDIT4* expression. Mechanistically, *DDIT4-AS1* formed an RNA duplex with *DDIT4* mRNA and enhanced its stability. Our explanation is that mRNA undergoes endonucleolytic or exonucleolytic degradation by various RNases; however, the RNA duplex formation could protect mRNAs from RNases degradation, thereby promoting mRNA stability [40]. A growing body of evidence suggests that antisense lncRNAs play important regulatory roles by forming RNA duplexes with mRNAs [23, 41–46]. A well-known case is *BACE-AS1*, which is transcribed from the opposite strand of *BACE1*. *BACE-AS1* forms an RNA duplex with *BACE1* and drives rapid feed-forward regulation of β-secretase [23]. In gastric cancer (GC), lncRNA *KRT7-AS* shows concordant expression with *KRT7* in GC tissues and cell lines. *KRT7-AS* increases *KRT7* mRNA stability by forming an RNA duplex, which promotes GC cell proliferation and migration [41]. In lung adenocarcinoma, lncRNA *MUC5B-AS1* promotes cell migration and invasion by increasing the stability of *MUC5B* mRNA through a mechanism involving RNA duplex formation [42]. In addition, the formation of an RNA duplex by antisense lncRNA and mRNA might cover microRNA binding sites of the mRNA, thereby stabilizing the mRNA [47, 48], which generally occurs in cytoplasm-enriched lncRNAs. Nucleus-enriched antisense lncRNAs can promote mRNA stability by modulating the association of RNA-binding proteins [43, 44]. For example, antisense lncRNA *Safe* could form an RNA duplex with *Sfrp2*, and the RNA binding protein HuR could bind to the *Safe*–*Sfrp2* RNA duplex and stabilize both *Safe* and *Sfrp2* [44]. Our RNA FISH assays demonstrated that *DDIT4-AS1* was located in cytoplasm and *DDIT4-AS1* co-located with *DDIT4*. Further experiments showed that *DDIT4-AS1* directly interacted with *DDIT4* mRNA and protected it from degradation. Moreover, the overexpression of *DDIT4-AS1* promoted the stability of *DDIT4* mRNA after treatment with α-amanitin. Our studies, in conjunction with these previous findings, suggest that antisense lncRNAs can modulate their sense mRNAs by forming RNA duplexes.

*DDIT4* has emerged as an important regulator of inflammatory responses. In a mouse model of cigarette smoke, *DDIT4* is determined to be upregulated in the lungs and forcefully expressing *DDIT4* promotes NF-κB activation and further exacerbates alveolar inflammation. However, alveolar inflammation and lung injury are markedly abrogated in *DDIT4* knockout mice [17]. *DDIT4* also aggravates LPS-induced systemic inflammation in macrophages, and the inflammatory responses are attenuated by *DDIT4* knockdown and knockout [14, 19]. In agreement with previous studies, the absence of *DDIT4* in astrocytes also decreased *E. coli*-induced inflammation, which further verified the regulatory function of *DDIT4* in inflammation. *DDIT4* knockout markedly alleviated the production of pro-inflammatory cytokines through the NF-κB signaling pathway. Recent evidence suggests that *DDIT4* interacts with and sequesters IκBα, thus promoting IKK independent atypical NF-κB activation [14]. It is possible that *DDIT4* knockout abrogated the sequestration of IκBα, which led to the suppression of NF-kB signaling. However, the specific molecular mechanisms require further investigation.

## Supplementary Information

Below is the link to the electronic supplementary material.

Supplementary file1 (DOCX 466 KB)

Supplementary file2 (XLS 51 KB)

Supplementary file3 (XLS 36 KB)

## Data Availability

All data generated or analyzed during this study are included in this published article. Ethics Approval and Consent to Participate. Not applicable. Consent for Publication. Not applicable.

## References

[1] Kim KS (2010). Acute bacterial meningitis in infants and children. Lancet Infect Dis.

[2] Wang X, Maruvada R, Morris AJ, Liu JO, Wolfgang MJ, Baek DJ, Bittman R, Kim KS (2016). Sphingosine 1-phosphate activation of EGFR as a novel target for meningitic *Escherichia coli* penetration of the blood-brain barrier. PLoS Pathog.

[3] Kim KS (2003). Pathogenesis of bacterial meningitis: from bacteraemia to neuronal injury. Nat Rev Neurosci.

[4] Kim KS, Itabashi H, Gemski P, Sadoff J, Warren RL, Cross AS (1992). The K1 capsule is the critical determinant in the development of *Escherichia coli* meningitis in the rat. J Clin Invest.

[5] Zhao WD, Liu DX, Wei JY, Miao ZW, Zhang K, Su ZK, Zhang XW, Li Q, Fang WG, Qin XX, Shang DS, Li B, Li QC, Cao L, Kim KS, Chen YH (2018). Caspr1 is a host receptor for meningitis-causing *Escherichia coli*. Nat Commun.

[6] Yang R, Liu W, Miao L, Yang X, Fu J, Dou B, Cai A, Zong X, Tan C, Chen H, Wang X (2016). Induction of VEGFA and Snail-1 by meningitic *Escherichia coli* mediates disruption of the blood-brain barrier. Oncotarget.

[7] Yang B, Yang R, Xu B, Fu J, Qu X, Li L, Dai M, Tan C, Chen H, Wang X (2021). miR-155 and miR-146a collectively regulate meningitic *Escherichia coli* infection-mediated neuroinflammatory responses. J Neuroinflammation.

[8] Ponting CP, Oliver PL, Reik W (2009). Evolution and functions of long noncoding RNAs. Cell.

[9] Chen YG, Satpathy AT, Chang HY (2017). Gene regulation in the immune system by long noncoding RNAs. Nat Immunol.

[10] Beermann J, Piccoli MT, Viereck J, Thum T (2016). Non-coding RNAs in development and disease: background, mechanisms, and therapeutic approaches. Physiol Rev.

[11] Kopp F, Mendell JT (2018). Functional classification and experimental dissection of long noncoding RNAs. Cell.

[12] Long Y, Wang X, Youmans DT, Cech TR (2017). How do lncRNAs regulate transcription?. Sci Adv.

[13] Tirado-Hurtado I, Fajardo W, Pinto JA (2018). DNA damage inducible transcript 4 gene: the switch of the metabolism as potential target in cancer. Front Oncol.

[14] Lee DK, Kim JH, Kim J, Choi S, Park M, Park W, Kim S, Lee KS, Kim T, Jung J, Choi YK, Ha KS, Won MH, Billiar TR, Kwon YG, Kim YM (2018). REDD-1 aggravates endotoxin-induced inflammation via atypical NF-kappaB activation. FASEB J.

[15] Li B, Chen R, Chen L, Qiu P, Ai X, Huang E, Huang W, Chen C, Liu C, Lin Z, Xie WB, Wang H (2017). Effects of DDIT4 in methamphetamine-induced autophagy and apoptosis in dopaminergic neurons. Mol Neurobiol.

[16] Brugarolas J, Lei K, Hurley RL, Manning BD, Reiling JH, Hafen E, Witters LA, Ellisen LW, Kaelin WG (2004). Regulation of mTOR function in response to hypoxia by REDD1 and the TSC1/TSC2 tumor suppressor complex. Genes Dev.

[17] Yoshida T, Mett I, Bhunia AK, Bowman J, Perez M, Zhang L, Gandjeva A, Zhen L, Chukwueke U, Mao T, Richter A, Brown E, Ashush H, Notkin N, Gelfand A, Thimmulappa RK, Rangasamy T, Sussan T, Cosgrove G, Mouded M, Shapiro SD, Petrache I, Biswal S, Feinstein E, Tuder RM (2010). Rtp801, a suppressor of mTOR signaling, is an essential mediator of cigarette smoke-induced pulmonary injury and emphysema. Nat Med.

[18] Nadon AM, Perez MJ, Hernandez-Saavedra D, Smith LP, Yang Y, Sanders LA, Gandjeva A, Chabon J, Koyanagi DE, Graham BB, Tuder RM, Schmidt EP (2014). Rtp801 suppression of epithelial mTORC1 augments endotoxin-induced lung inflammation. Am J Pathol.

[19] Pastor F, Dumas K, Barthelemy MA, Regazzetti C, Druelle N, Peraldi P, Cormont M, Tanti JF, Giorgetti-Peraldi S (2017). Implication of REDD1 in the activation of inflammatory pathways. Sci Rep.

[20] Angelidou I, Chrysanthopoulou A, Mitsios A, Arelaki S, Arampatzioglou A, Kambas K, Ritis D, Tsironidou V, Moschos I, Dalla V, Stakos D, Kouklakis G, Mitroulis I, Ritis K, Skendros P (2018). REDD1/autophagy pathway is associated with neutrophil-driven IL-1beta inflammatory response in active ulcerative colitis. J Immunol.

[21] Liu C, Zheng H, Yang M, Xu Z, Wang X, Wei L, Tang B, Liu F, Zhang Y, Ding Y, Tang X, Wu B, Johnson TJ, Chen H, Tan C (2015). Genome analysis and in vivo virulence of porcine extraintestinal pathogenic *Escherichia coli* strain PCN033. BMC Genomics.

[22] Castanotto D, Lingeman R, Riggs AD, Rossi JJ (2009). CRM1 mediates nuclear-cytoplasmic shuttling of mature microRNAs. Proc Natl Acad Sci U S A.

[23] Faghihi MA, Modarresi F, Khalil AM, Wood DE, Sahagan BG, Morgan TE, Finch CE, St-Laurent G, Kenny PJ, Wahlestedt C (2008). Expression of a noncoding RNA is elevated in Alzheimer’s disease and drives rapid feed-forward regulation of beta-secretase. Nat Med.

[24] Yang B, Yin P, Yang R, Xu B, Fu J, Zhi S, Dai M, Tan C, Chen H, Wang X (2020). Holistic insights into meningitic *Escherichia coli* infection of astrocytes based on whole transcriptome profiling. Epigenomics.

[25] Faghihi MA, Wahlestedt C (2009). Regulatory roles of natural antisense transcripts. Nat Rev Mol Cell Biol.

[26] Pelechano V, Steinmetz LM (2013). Gene regulation by antisense transcription. Nat Rev Genet.

[27] Sun L, Luo H, Bu D, Zhao G, Yu K, Zhang C, Liu Y, Chen R, Zhao Y (2013). Utilizing sequence intrinsic composition to classify protein-coding and long non-coding transcripts. Nucleic Acids Res.

[28] Kong L, Zhang Y, Ye ZQ, Liu XQ, Zhao SQ, Wei L, Gao G (2007). CPC: assess the protein-coding potential of transcripts using sequence features and support vector machine. Nucleic Acids Res.

[29] Li A, Zhang J, Zhou Z (2014). PLEK: a tool for predicting long non-coding RNAs and messenger RNAs based on an improved k-mer scheme. BMC Bioinformatics.

[30] Atianand MK, Caffrey DR, Fitzgerald KA (2017). Immunobiology of long noncoding RNAs. Annu Rev Immunol.

[31] Villegas VE, Zaphiropoulos PG (2015). Neighboring gene regulation by antisense long non-coding RNAs. Int J Mol Sci.

[32] Wahlestedt C (2006). Natural antisense and noncoding RNA transcripts as potential drug targets. Drug Discov Today.

[33] Meng W, Cui W, Zhao L, Chi W, Cao H, Wang B (2019). Aberrant methylation and downregulation of ZNF667-AS1 and ZNF667 promote the malignant progression of laryngeal squamous cell carcinoma. J Biomed Sci.

[34] Gomez JA, Wapinski OL, Yang YW, Bureau JF, Gopinath S, Monack DM, Chang HY, Brahic M, Kirkegaard K (2013). The NeST long ncRNA controls microbial susceptibility and epigenetic activation of the interferon-gamma locus. Cell.

[35] Zhu H, Wang Q, Yao Y, Fang J, Sun F, Ni Y, Shen Y, Wang H, Shao S (2015). Microarray analysis of long non-coding RNA expression profiles in human gastric cells and tissues with *Helicobacter pylori* infection. BMC Med Genomics.

[36] Wang Y, Zhong H, Xie X, Chen CY, Huang D, Shen L, Zhang H, Chen ZW, Zeng G (2015). Long noncoding RNA derived from CD244 signaling epigenetically controls CD8+ T-cell immune responses in tuberculosis infection. Proc Natl Acad Sci U S A.

[37] Yi Z, Li J, Gao K, Fu Y (2014). Identification of differentially expressed long non-coding RNAs in CD4+ T cells response to latent tuberculosis infection. J Infect.

[38] Chan J, Atianand M, Jiang Z, Carpenter S, Aiello D, Elling R, Fitzgerald KA, Caffrey DR (2015). Cutting edge: a natural antisense transcript, AS-IL1alpha, controls inducible transcription of the proinflammatory cytokine IL-1alpha. J Immunol.

[39] Atianand MK, Hu W, Satpathy AT, Shen Y, Ricci EP, Alvarez-Dominguez JR, Bhatta A, Schattgen SA, McGowan JD, Blin J, Braun JE, Gandhi P, Moore MJ, Chang HY, Lodish HF, Caffrey DR, Fitzgerald KA (2016). A long noncoding RNA lincRNA-EPS acts as a transcriptional brake to restrain inflammation. Cell.

[40] Stazic D, Lindell D, Steglich C (2011). Antisense RNA protects mRNA from RNase E degradation by RNA-RNA duplex formation during phage infection. Nucleic Acids Res.

[41] Huang B, Song JH, Cheng Y, Abraham JM, Ibrahim S, Sun Z, Ke X, Meltzer SJ (2016). Long non-coding antisense RNA KRT7-AS is activated in gastric cancers and supports cancer cell progression by increasing KRT7 expression. Oncogene.

[42] Yuan S, Liu Q, Hu Z, Zhou Z, Wang G, Li C, Xie W, Meng G, Xiang Y, Wu N, Wu L, Yu Z, Bai L, Li Y (2018). Long non-coding RNA MUC5B-AS1 promotes metastasis through mutually regulating MUC5B expression in lung adenocarcinoma. Cell Death Dis.

[43] Jadaliha M, Gholamalamdari O, Tang W, Zhang Y, Petracovici A, Hao Q, Tariq A, Kim TG, Holton SE, Singh DK, Li XL, Freier SM, Ambs S, Bhargava R, Lal A, Prasanth SG, Ma J, Prasanth KV (2018). A natural antisense lncRNA controls breast cancer progression by promoting tumor suppressor gene mRNA stability. PLoS Genet.

[44] Hao K, Lei W, Wu H, Wu J, Yang Z, Yan S, Lu XA, Li J, Xia X, Han X, Deng W, Zhong G, Zhao ZA, Hu S (2019). LncRNA-Safe contributes to cardiac fibrosis through Safe-Sfrp2-HuR complex in mouse myocardial infarction. Theranostics.

[45] Guo W, Liu S, Cheng Y, Lu L, Shi J, Xu G, Li N, Cheng K, Wu M, Cheng S, Liu S (2016). ICAM-1-related noncoding RNA in cancer stem cells maintains ICAM-1 expression in hepatocellular carcinoma. Clin Cancer Res.

[46] Michael DR, Phillips AO, Krupa A, Martin J, Redman JE, Altaher A, Neville RD, Webber J, Kim MY, Bowen T (2011). The human hyaluronan synthase 2 (HAS2) gene and its natural antisense RNA exhibit coordinated expression in the renal proximal tubular epithelial cell. J Biol Chem.

[47] Faghihi MA, Zhang M, Huang J, Modarresi F, Van der Brug MP, Nalls MA, Cookson MR, St-Laurent G, Wahlestedt C (2010). Evidence for natural antisense transcript-mediated inhibition of microRNA function. Genome Biol.

[48] Wang GQ, Wang Y, Xiong Y, Chen XC, Ma ML, Cai R, Gao Y, Sun YM, Yang GS, Pang WJ (2016). Sirt1 AS lncRNA interacts with its mRNA to inhibit muscle formation by attenuating function of miR-34a. Sci Rep.

