# Supplementary material for: Long Non-coding Antisense RNA DDIT4-AS1 Regulates Meningitic Escherichia coli-Induced Neuroinflammation by Promoting DDIT4 mRNA Stability
Source: Mol Neurobiol. 2022 Jan 5;59(3):1351–65. doi: 10.1007/s12035-021-02690-6 (PMC8882120; doi:10.1007/s12035-021-02690-6)
Supplement: Supplementary file 1 — Supplementary file1 (DOCX 466 KB) [file 12035_2021_2690_MOESM1_ESM.docx]

**Supplementary Materials**

**Table S1**. Primers used in this study

| Gene | Forward (5′–3′) | Reverse (5′–3′) |
| --- | --- | --- |
| GAPDH  DDIT-AS1  DDIT4  RP11-796E2.4  BTG1  RP11-809N8.2  RELT  RP4-781K5.2  IRF2BP2  CTD-2540B15.11  CEBPA  RP11-1143G9.4  LYZ | TGCCTCCTGCACCACCAACT  CTACAACAGGTCATAACAAAAAT  CCACCTCCTCTTCGCCCTC  CTGGAGTGTAAGTGCTGGGTA  CCACCATGATAGGCGAGATCG  GTGCCCAAGATTAGAACCC  GTTCCATGTCAACCATGTTCCT  TCCAGTCCTCCTGTGTTG  GGCAGGTTGTTGGGTTTCG  AAATGGTGGTTTAGCAGA  CGTGGAGACGCAGCAGAAGG  GCTGGTATTTTGCCTAAG  TCAATAGCCGCTACTGGTGTA | CGCCTGCTTCACCACCTTC  ATGAAACAAAGGCTTAGGG  AGCCACTGTTGCTGCTGTCC  GAGCGGGAACGAGATGTA  GGTTGATGCGAATACAACGGTA  CCCGTGCCTCTGTACAGG  AGGCAGAAGACAGGGACGAT  CCTCTCCGTCCTCTATGAAC  GAGCCCCTCTGTGGATGTGG  AACCTTGTGCCTAGGAACAC  CCGCAGCGTGTCCAGTTCG  TGCCTCAGCCTCCCAAAG  ATCACGGACAACCCTCTTTGC |
| 18S  U6 | CGGACAGGATTGACAGATTGATAGC  CTCGCTTCGGCAGCACA | TGCCAGAGTCTCGTTCGTTATCG  AACGCTTCACGAATTTGCGT |
| IL1β | ATGATGGCTTATTACAGTGGCAA | GTCGGAGATTCGTAGCTGGA |
| TNF-α  DDIT4-sgRNA1  DDIT4-sgRNA2  DDIT4-OL  DDIT4-NON-OL  pEGFP-mut | CGAGTGACAAGCCTGTAG  CACCGTGAGCGCGGCGGCCGATCTG  CACCGTCCGAGCTCTCCAGGCTCG  CGTGGAGCAGGGCAAGAG  ACCTCCTCTTCGCCCTCG  GCCACCATTGTTAGCAAGGGCGAGGAGCTG | GGACCTGGGAGTAGATGA  AAACCAGATCGGCCGCCGCGCTCAC  AAACCGAGCCTGGAGAGCTCGGAC  GCCAGGGAGGAAGGGAGA  GTCAAACCCCTCCTCCCG  CTTGCTAACAATGGTGGCGACCGGTGGATC |

**Table S2**. Probes and ASOs used in this study

| Name | Sequence (5′–3′) |
| --- | --- |
| DDIT4 FISH probe  DDIT4-AS1 FISH probe  DDIT4-AS1 RAP probe1  DDIT4-AS1 RAP probe2  DDIT4-AS1 RAP probe3  DDIT4-AS1 RAP probe4  DDIT4-AS1 RAP probe5  NC-ASO  DDIT4-AS1-ASO | TGCTGATGAACTCAGAGTGC ACCTTCCAGCTGACCCTCGT  ATCGTGTACTGCAGAGTTGA  CGCATGAATGTAAGAGTAGG  AACAAAGGCTTAGGGGCCAA  TAAGGTGGAGGTGGGGGAAT  TTGAACTTCAACCTGAGGGG  CCTTCCCTGAAGGTTCCTCC  AAGAAGAAGCTGTACAGCTC |

**Table S3**. The sequence of *DDIT4-AS1* and potential ORF of *DDIT4-AS1*

| Name | Sequence |
| --- | --- |
| DDIT4-AS1  potential ORF of DDIT4-AS1 | TTGAACATCAAGTGTATTCATGAACAGTGAGTATCTTATCTTCATGTAAACAGTTCTAGATGGAAGACCCAGATGGCACTCCTCCCGGGGAGGGGTTCCAGCCCCCACCCTCTCAGCCCCTCCCCTGCCAGCTCAACTCTGCAGTACACGATGGGGGAAGGCTTAAACGCAGCTGCCAGGTGTAATTTTTCAAGTGTCAAAGATCCCAAGTGATCCCTGACACCCACCCCTTCCTACTCTTACATTCATGCGTCTGTAAGATAGCTGCCTACAACAGGTCATAACAAAAATAATAAGGTACATGCTACACACACATCCAGCTGGAAGCCTTGTTGGCCCCTAAGCCTTTGTTTCATGCTACAGTACTGAGGGGTATGTGTCCCCAATGCACAGCCACCCGCACACAACTCAATGAGCTTCCTGGGAAACACTATTCCCCCACCTCCACCTTAGGTGGCTGCCTCAGTTTTCCAACCACAGGAATCAGTCCCTCAGCTCCTGCCTCTAGTCTCCACCCCAAAAGTTCAGTCGTCTCTGTCTTGGAGGGCACTGTCGGCCCCCTCAGGTTGAAGTTCAACACTCCTCAATGAGCAGCTGTTCCGAGCTGTACAGCTTCTTCTTGATGACTCGGAAGCCAGTGCTCAGCGTCAGGGACTGGCTGAAGCCAGGGAGGAAGGGAGAGTTGGCGGAGCTAAACAGCCCCTGGATCTTGGGCCAGAGTCGTGAGTCCAGGCGCAGCACGAGGGTCAGCTGGAAGGTGGGCACCAGGCTGGGGTCGAGTGCCAGCTGGCCCACGCTGTGGCAGCTCTTGCCCTGCTCCACGCAGACGTCCAGCAGCGCCCCCCGC  ATGGCACTCCTCCCGGGGAGGGGTTCCAGCCCCCACCCTCTCAGCCCCTCCCCTGCCAGCTCAACTCTGCAGTACACGATGGGGGAAGGCTTAAACGCAGCTGCCAGGTGTAATTTTTCAAGTGTCAAAGATCCCAAGTGA |


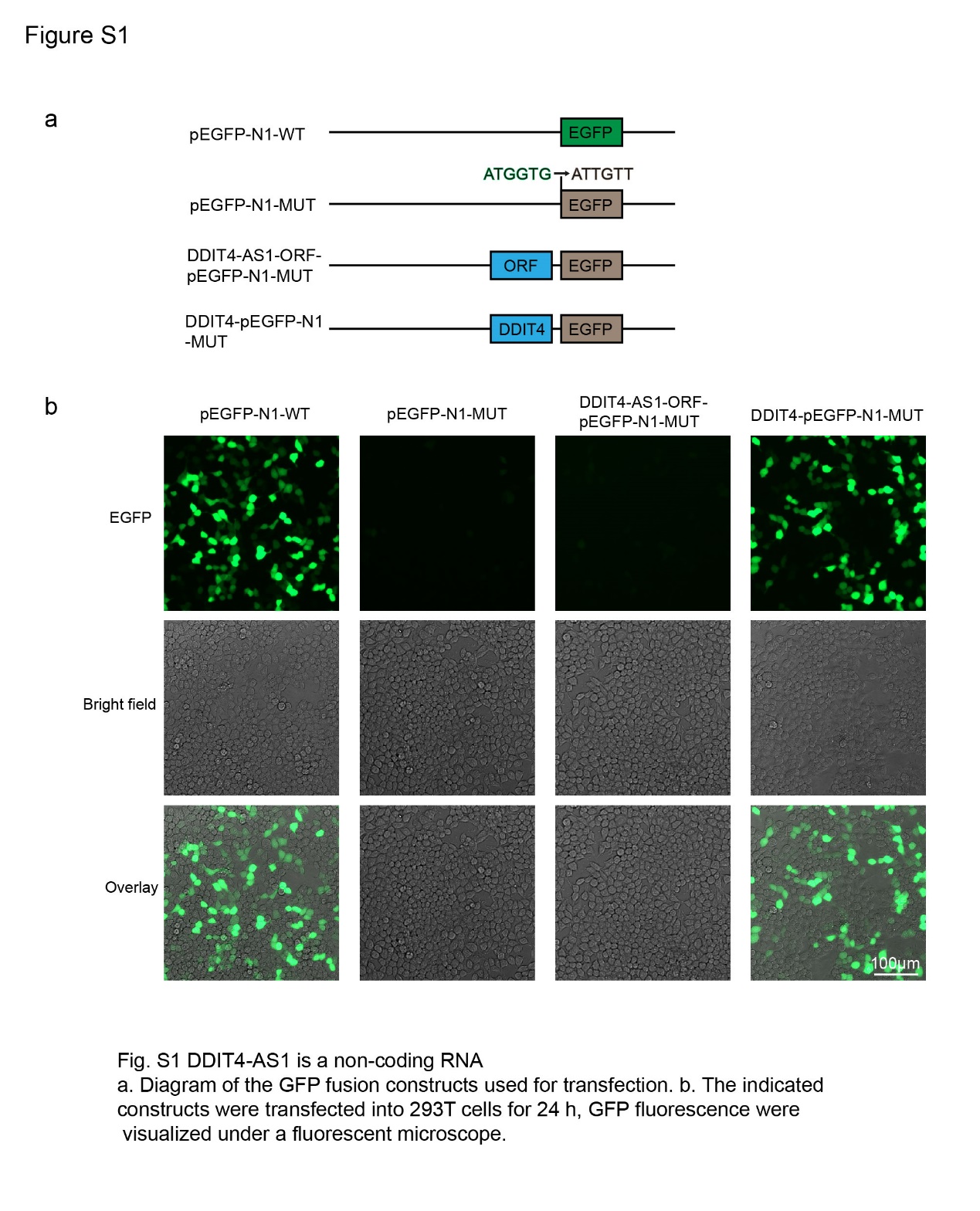


Fig. S1 DDIT4-AS1 is a non-coding RNA

a. Diagram of the GFP fusion constructs used for transfection. b. The indicated constructs were transfected into 293T cells for 24 h, GFP fluorescence were visualized under a fluorescent microscope.
